# The Emergence of a Phoneme-Sized Unit in L2 Speech Production: Evidence from Japanese–English Bilinguals

**DOI:** 10.3389/fpsyg.2016.00175

**Published:** 2016-02-23

**Authors:** Mariko Nakayama, Sachiko Kinoshita, Rinus G. Verdonschot

**Affiliations:** ^1^Faculty of Letters, Arts and Sciences, Waseda UniversityTokyo, Japan; ^2^ARC Centre of Excellence in Cognition and its Disorders, SydneyNSW, Australia; ^3^Department of Psychology, Macquarie University, SydneyNSW, Australia; ^4^Waseda Institute for Advanced StudyTokyo, Japan

**Keywords:** language production, masked priming, phonological unit, proximate unit, Japanese, bilingualism

## Abstract

Recent research has revealed that the way phonology is constructed during word production differs across languages. Dutch and English native speakers are suggested to incrementally insert *phonemes* into a metrical frame, whereas Mandarin Chinese speakers use syllables and Japanese speakers use a unit called the mora (often a CV cluster such as “ka” or “ki”). The present study is concerned with the question how *bilinguals* construct phonology in their L2 when the phonological unit size differs from the unit in their L1. Japanese–English bilinguals of varying proficiency read aloud English words preceded by masked primes that overlapped in just the onset (e.g., bark-BENCH) or the onset plus vowel corresponding to the mora-sized unit (e.g., bell-BENCH). Low-proficient Japanese–English bilinguals showed CV priming but did *not* show onset priming, indicating that they use their L1 phonological unit when reading L2 English words. In contrast, high-proficient Japanese–English bilinguals showed significant onset priming. The size of the onset priming effect was correlated with the length of time spent in English-speaking countries, which suggests that extensive exposure to L2 phonology may play a key role in the emergence of a language-specific phonological unit in L2 word production.

## Introduction

Speaking a word naturally requires assembling its phonology. According to the influential language production model by Levelt et al. (1999), this takes place through a process called *prosodification*. This entails first accessing a word’s *phonological segments* (e.g., phonemes in English/Dutch), which are then incrementally inserted into a metrical frame (a structure specifying the number of syllables and the stress position). That is, producing a word such as “table” in English will first require access to its phonemes (i.e., /t/ /e/ /I/ /b/ /ə/ /l/) and metrical structure (i.e., bi-syllabic with stress on first syllable) which are then merged together to form the phonological word (i.e., /ter’-bəl/). Constructing phonology on-line is essential for languages such as English and Dutch (on which the Levelt et al. (1999) model is mainly based) as these languages often need re-syllabification depending on the local context. For instance, the sentence “He’ll escort us.” is normally pronounced as /hil-ə-skɔr–təs/. As the cliticized form (/ə-skɔr-təs/) would not be stored in the lexicon, whether the syllable /-skɔr’/ or /-skɔrt’/ will be created depends on the utterance context (Levelt et al., 1999, p. 23). The evidence that this process initially occurs in phoneme-sized units comes from results obtained in Dutch using the implicit priming (also called the form preparation) paradigm (Meyer, 1990, 1991). In this paradigm, participants learn prompt-response pairs (e.g., say “DANS” [*dance*] when presented with the prompt “feest" [party]). The prompted words are grouped in such a way that they either all overlapped in their initial segment(s) or not. Response words were produced significantly faster when there was overlap (e.g., DANS [dance], DOP [cap], DEUGD [virtue]) compared to when there was no overlap (e.g., DANS [dance], HEKS [witch], STOEP [sidewalk]). This significant facilitation is referred to as *the preparation effect*. In contrast, rime related overlap (e.g., BOEK [book], DOEK [canvas], SNOEK [pike]) did not produce facilitation, attesting to the incremental left-to-right (i.e., beginning to end) nature of the segment-to-frame association process.

Research on reading aloud has also revealed a similar left-to-right incremental segment-to-frame association process. Masked priming research using English (e.g., Forster and Davis, 1991; Kinoshita, 2000) and Dutch (e.g., Schiller, 2004) has also shown that when a prime is briefly presented (e.g., 50 ms) before a to-be-read-aloud target, naming latencies are significantly faster when the onset phoneme is shared (e.g., pole-PEAR) than when it is not (e.g., take-PEAR). Similar to findings observed with the implicit priming paradigm, no facilitation was observed in masked priming when only the last segments were shared (e.g., Kinoshita, 2000; Schiller, 2008). While the masked onset priming effect was originally interpreted in terms of a serial *letter-to-phoneme mapping process* (e.g., Forster and Davis, 1991), the emerging consensus is that this left-to-right incremental nature of reading reflects a speech production process (see e.g., Grainger and Ferrand, 1996; Roelofs, 2004; Malouf and Kinoshita, 2007).

The evidence for the left-to-right phoneme-to-frame association process mentioned above has come from European languages, mainly Dutch and English. However, languages differ in many respects, and recently it has been suggested that the unit used to fill the metrical frame may not always be the phoneme, but other languages may employ different unit sizes (see O’Seaghdha, 2015; Roelofs, 2015). For instance, in Mandarin Chinese (hereafter “Chinese”), Chen et al. (2002) and O’Seaghdha et al. (2010) employing the implicit priming paradigm, found reliable preparation effects only when a group of response words overlapped in the first (atonal) *syllable*; no facilitation was observed when a group of response words overlapped in the onset phoneme. The initial unit involved to build phonology (termed the “proximate unit” by O’Seaghdha et al., 2010) in Chinese, therefore, seems to be the syllable, and not the phoneme (see also You et al., 2012, for related results).

### The Proximate Unit of Japanese Word Production

Japanese is known to have a mora-based timing (Warner and Arai, 2001; Kureta et al., 2006). The Japanese mora is a supra phonemic unit that usually involves a CV or V (e.g., /ka/ or /a/), nasal coda (/N/), or a geminate (/Q/) combination, but *never* a single consonant (e.g., /k/). The mora as a proximate unit has accounted for many Japanese psycholinguistic findings ranging from speech segmentation (e.g., Cutler and Otake, 1994), speech errors (e.g., Kubozono, 1989), and children’s word games (e.g., “shiritori,” in which the players take turns in generating a word that starts with the final mora of the word the other player has produced: e.g., “kobuta” (piglet) – “tanuki”(badger)” – “kitsune” (fox) – “neko” (cat) and so on, see e.g., Katada, 1990). Phonological awareness tests typically assess skills of mora level manipulation, not phonemes (e.g., Sasanuma et al., 1996). The central importance of the mora as *the* phonological unit in Japanese is further evidenced in the phenomenon of “vowel epenthesis,” a form of phonological restoration: When presented with a non-word containing an illegal consonant cluster like “ebzo,” Japanese listeners hear an illusory vowel, reporting they heard “ebuzo” (Dupoux et al., 2001). Moreover, Japanese listeners show no mismatch negativity in evoked potentials to a change from “ebzo” to “ebuzo,” whereas French listeners do (Dehaene-Lambertz et al., 2000). Additionally, when producing English words, Japanese people typically insert vowels when a word contains phoneme clusters (Broselow and Park, 1995; Broselow and Kang, 2013).

Previous studies on word production also indicate the critical role of the mora during Japanese phonological encoding. Kureta et al. (2006) using the implicit priming paradigm found significant preparation effects in Japanese *only* when a group of response words overlapped in the initial mora, but *not* when they merely overlapped in the onset phoneme. Using a masked priming read-aloud paradigm, Verdonschot et al. (2011) reported that Japanese words were read aloud significantly faster when a target was preceded by a prime overlapping in the initial mora (e.g., teki-TENSHI) relative to unrelated primes (e.g., heki-TENSHI). Critically, reading of the Japanese words *never* benefited from a prime overlapping in the onset phoneme (e.g., tomi-TENSHI) relative to a control prime (e.g., gomi-TENSHI).

One important point in interpreting the masked onset priming effect is the role of script. Indo-European languages like English and Dutch use the alphabetic writing system, in which a letter (or letter cluster e.g., “sh”) maps onto a phoneme. Chinese is written using a logography in which a character maps onto a (morpho-) syllable. Japanese is written both in “kanji” (literally “Chinese characters”), the logography borrowed from the Chinese, and in “kana” (hiragana/katakana), two inventories, consisting of 48 characters each, mapping onto a mora (e.g., 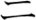 [ni] • 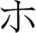 [ho] • 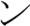 [N] and 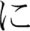 [ni] • 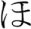 [ho] • 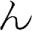 [N], for katakana and hiragana, respectively).^1^ In the masked priming read-aloud experiments mentioned earlier, all words were presented in their native script, i.e., alphabetic letters in English (e.g., Kinoshita, 2000) and Dutch (e.g., Schiller, 2004) and kana in Japanese (Verdonschot et al., 2011). As noted earlier, an alternative interpretation of the masked onset priming effect in reading-aloud is that it might originate in the mapping of letters to phonemes (Forster and Davis, 1991). From this perspective, the absence of masked onset priming effect in studies that used non-alphabetic script like kana may be interpreted as reflecting the size of the unit involved in the mapping of written script to phonology, rather than the size of the phonological unit involved in speech production. To test this possibility, Verdonschot et al. (2011) conducted two experiments in which Japanese target words were presented in “romaji” (alphabetic transcriptions). However, no significant onset priming effect was found in either experiment, suggesting that for the Japanese speakers, the effect depended on the size of the phonological unit used in speech production rather than print-to-speech conversion.

### Phonological Units in L2 Word Production

Given the increasing evidence concerning the difference between languages in the proximate unit (the primary unit used in the phonological encoding process), the logical next step is to investigate how *bilinguals* process words, and how L2 proficiency modulates this process. Not surprisingly, the earlier L2 is acquired, the more native-like the bilingual speakers’ pronunciation of L2 becomes (see Piske et al., 2001, for a review). As noted by Alario et al. (2010) however, most of research on this issue has focused on the acoustic properties of bilinguals’ speech, and studies focusing on the cognitive mechanisms involved in the spoken production of L2 are very scarce. In particular, it is currently unknown what phonological unit is used in L2 production when bilinguals’ two languages have different unit sizes.

To our knowledge, only one study to date has investigated this matter (Verdonschot et al., 2013). That study involved highly proficient Chinese-English bilinguals to read aloud English targets primed by English words. Naming latency was significantly faster when a target was primed by an onset-related English word (e.g., bark – BENCH) than by an unrelated prime (e.g., dark-BENCH). As noted earlier, the phonological unit of monolingual Chinese speakers is known to be a syllable (e.g., CVC). Therefore, the significant onset priming observed for Chinese-English bilinguals suggests that highly proficient bilinguals used a phonological unit suited to produce L2 words (i.e., phonemes), one that is different in size from the phonological unit normally used in their L1 production (i.e., syllable).

A possible limitation concerning Verdonschot et al. (2013) is that the Chinese-English bilinguals were all highly proficient. It is therefore unknown whether the ability to prepare phonology in the unit of L2 develops with proficiency in L2. Also, Verdonschot et al. (2013) did not include a group of native English speakers. Therefore, it would be important to show that the high-proficient bilinguals behave more like native English speakers than the low-proficient bilinguals in producing a significant onset priming effect with the same set of stimuli.

### Present Study

The present study investigated the proximate unit used by Japanese–English bilinguals of varying proficiency in reading aloud L2 (English) words. Specifically, we were interested in whether the L1 Japanese speaker constructs L2 English phonology by placing moras (CVs) or phonemes (specifically consonants, given that a vowel is also a mora) in the metrical frame, and whether the size of the phonological unit is modulated by L2 proficiency. To assess this, low-proficient bilinguals, high-proficient bilinguals and native English monolingual speakers (Experiments 1–3, respectively) read aloud English target words that were preceded either by English prime words that shared the initial onset phoneme (bark-BENCH) or by words that shared the initial CV (i.e., mora; bell-BENCH), with priming effects measured against their respective unrelated primes (dark-BENCH and cell-BENCH). Assuming that the low-proficient Japanese–English bilinguals would use the phonological unit of their first language (the mora), they should show *CV (mora) priming* effects (bell-BENCH < cell-BENCH), but not *onset priming* (phoneme) effects (bark-BENCH = dark-BENCH). Alternatively, if a significant onset effect *is* observed for low-proficient bilinguals, this would then suggest that the proximate unit of L2 English (phoneme) can be adopted relatively early in the course of L2 acquisition. In contrast, high-proficient bilinguals are more likely to show onset effects, based on the finding by Verdonschot et al. (2013) with high-proficiency Chinese–English bilinguals. If so, this would extrapolate previous findings (L1-Chinese vs. L2-English) to a group of bilinguals whose two proximate units also diverge in their two languages (L1-Japanese vs. L2-English). Finally, we expect the group of native English speakers to show significant onset priming effects, in line with previous studies (Forster and Davis, 1991; Kinoshita, 2000; Schiller, 2004).

## Experiment 1: Low-Proficient Japanese–English Bilinguals

### Methods

#### Participants

Forty-five low proficient Japanese–English bilingual students from Waseda University (Tokyo, Japan) participated in the experiment in return for payment of 1000 Yen (∼US$8). Their mean TOEIC (Test of English for International Communication) score was 715 (range = 600–790).^2^ This study was carried out in accordance with the recommendations of ‘*the Ethics Guidelines for Scientific Research with Human Subjects, Ethics Review Committee on Research of Waseda University’* and *‘the Human Research Ethics Committee of Macquarie University.’* Prior to the experiments, all subjects gave written informed consent in accordance with the Declaration of Helsinki.

#### Stimuli

The critical stimuli were 42 English medium frequency words (*M* = 50.3 occurrences per million, Kućera and Francis, 1967). The mean letter length and syllable size of the targets were 5.1 (*SD* = 0.9) and 1.5 syllables. The syllable length was equally distributed between one (*n* = 21) or two syllables (*n* = 21). For each target, four types of monosyllabic English word primes were selected: (1) C prime: a word that had the same onset phoneme with the target (e.g., bark-BENCH), (2) C-control prime: a word that shared all the letters with the onset prime except for the initial letter (e.g., dark-BENCH), (3) CV prime: a word that had the same CV with the target (e.g., bell-BENCH) and (4) CV-control prime: a word that shared all the letters with the CV prime except for the initial letter (e.g., cell-BENCH).^3^ This ensured that CV prime-target pairs do not have an additional letter (and phoneme) overlap compared with C prime-target pairs (e.g., Verdonschot et al., 2011, 2013). In addition, the bodies of C/CV primes and their controls always had the same pronunciation (e.g., -ark in “bark – dark” or –ell in “bell – cell”). The mean word frequencies (per million) of the four types of primes (C, C-control, CV, CV-control) were comparable: 52.8, 59.4, 58.2, and 50.9, respectively. The word lengths (in letters) of the four types of primes were also comparable (3.6, 3.6, 3.8, and 3.8). For the C and CV conditions, there were two counterbalanced lists; within each condition, half of the targets were primed by the critical primes in one list, and the same targets were primed by their control primes in the other lists, and vice versa. The list of prime and target stimuli used can be found in the Supplementary Materials.

To check the possibility that an absence of masked priming might be due to the lack of familiarity with the alphabetic letters, an identity priming condition was included (and also in subsequent two experiments). The masked identity priming effect is known to be unaffected by word frequency (Forster and Davis, 1984) and it is generally interpreted to reflect a “head-start” in orthographic processing (Gomez et al., 2013). The presence of a typical identity priming effect (e.g., the sizes of priming effects being ±10 ms of the prime duration, see Forster et al., 2003) would indicate participants’ ability to process masked primes in alphabetic script.

For the identity priming condition, a different set of 42 medium frequency targets were selected (*M* = 83.2 per million). The mean length of these targets was 4.4 letters (60% consisted of one syllable, 40% consisted of two syllables). Each target (e.g., SOFT) was primed either by the target itself (i.e., soft) or by a control prime that did not share any letters with the target at the same position (e.g., page). The mean word frequency and the mean length of the control primes were 74.3 per million and 4.4 letters. None of the words in the identity priming condition were used in the C/CV conditions. For the identity priming condition, there also were two counterbalanced lists in order to present the same targets to all participants but each participant saw only one of the prime-target pairings.

### Apparatus and Procedure

Participants were tested individually using the DMDX software package (Forster and Forster, 2003). Each trial began with the presentation of a forward mask (#####) for 500 ms followed by a 50 ms presentation of a lower case prime. Immediately following the prime, a target was presented in upper case. The target remained on the display until the participant made a response. Participants were instructed to read aloud the target as quickly and accurately as possible. The stimuli were presented at the center of the screen in 12-pt Courier New font. The presence of primes was not mentioned to any participant. Participants completed 16 practice trials to familiarize themselves with the task.

For the C and CV priming conditions, the same set of 42 targets was presented twice, once in the C condition and once in the CV condition. The identity priming condition was always presented in between the C and CV conditions. Half of the participants were presented with the C condition in the first block, and the CV condition in the third block; the other half were presented with the CV condition in the first block, and the C condition in the third block. Targets primed by critical primes (either C or CV) in the first block were primed by control primes in the third block, and vice versa. Therefore, for the C/CV conditions, although there were two counterbalancing lists with regard to prime-target relationships (i.e., related vs. control), there were four presentation orders differing in whether the target was paired with an C prime or a CV prime first, crossed with the two lists.

### Results

Raw naming reaction times (RTs) were checked using CheckVocal Software (Protopapas, 2007). We used a linear mixed-effect (LME) model (lme4; Baayen, 2008; Bates et al., 2008) implemented in R (R Development Core Team, 2008) to analyze RT for correct trials and error rates. *lmerTest* package in R was used to calculate the *p*-values using Satterthwaite’s approximation for the degrees of freedom (Kuznetsova et al., 2014). In order to meet the distributional assumptions of LME, we applied the inverse transformation to the RTs (–1000/RT) to better approximate normality in the RT distribution (see Box and Cox, 1964). Correct data points that were 3.5 SD away from the individual’s mean per condition were removed as outliers (both 0.3% of the data in the C/CV conditions and Identity condition, respectively). In the identity priming condition, three items (DENY, TINY, RIFLE) were removed due to high error rates (>55%).

For the C and CV conditions, the initial model included Overlap (CV vs. C), Prime Type (related vs. control) and Order (first vs. third block) and their interaction as fixed factors, and by-subject intercept and slope and by-item intercept and slope of Overlap, Prime Type, and their interaction as random factors. Note that Block 2 is not considered in the Order variable as it always contained the identity primes. Each of the categorical variables was contrast coded by 0.5/-0.5. We also entered the following target lexical characteristics as fixed factors: Log subtlex frequency (Brysbaert and New, 2009), Orthographic neighborhood size (Ortho-N), and Length. These continuous variables were centered around their respective means. In addition, because Length and Ortho-N were moderately correlated (*r* = 0.53), Ortho-N was regressed against Length and their residuals were used as a predictor for Ortho_N (i.e., res_Ortho-N). Thus the model used in the analyses was [invRT ∼ Overlap^∗^PrimeType^∗^Order + Log subtlex frequency + res_Ortho-N + Length + (1 + Overlap^∗^ Prime Type| subject) + (1 + Overlap^∗^Prime Type| target)].

For the identity priming condition, the model used in the analyses was the same as above except that Order was not included as a factor. For the C, CV and Identity conditions, errors were analyzed using a mixed-effects logistic model (Jaeger, 2008) using the same fixted factors used for RT analyses.^4^ However, the error rates were small and there were no significant priming effects in any conditions except in the identity condition (*p* = 0.02), therefore we will only report the results of response latencies analyses. **Table 1** shows the mean RT and error rates for the three conditions. **Table 1** shows the mean RT and error rates for the three conditions.

**Table 1:** Mean naming latencies (ms) and percentage errors (%) for English targets primed by C primes, C control primes, CV primes, CV control primes, identity primes and identity control primes in Experiment 1, for low-proficient bilinguals.

	C condition	CV condition	Identity condition
Related prime	635 (5.2%)	627 (3.4%)	660 (6.1%)
Control prime	633 (5.4%)	652 (4.2%)	701 (8.6%)
Priming effect	–2 (0.2%)	25 (0.8%)	41 (2.5%)

#### Onset (C) and CV Priming Effects

Order did not significantly affect the patterns of priming as indicated by the lack of three-way interaction between Order, Overlap, Prime Type (*t* < 1) and also by the lack of two-way interaction between Order and Prime Type (*t* < 1). The main effect of Order was statistically significant (*t* = 5.10, *p* < 0.001); naming latencies were significantly faster in the third block than in the first block. The main effect of Prime Type was significant (*t* = 4.13, *p* < 0.001). The main effect of Overlap was not significant (*t* < 1). Importantly, there was a significant interaction between Overlap and Prime Type (*t* = 2.57, *p* = 0.014). Follow-up analyses of this interaction revealed that there was *no* C (onset) priming (*t* = 1.02, *p* > 0.10, a –2 ms difference; in contrast, there was a significant CV priming effect (*t* = 4.46, *p* < 0.001, a 25 ms effect). As for the effects of target lexical characteristics, there was a significant effect of Log subtlex frequency (*t* = –7.47, *p* < 0.001), Ortho-N (*t* = –3.76, *p* < 0.001), and Length (*t* = 4.23, *p* < 0.001), that is, faster naming latencies were associated with targets with higher frequency, more orthographic neighbors, and shorter lengths.

#### Identity Priming Effects

The effect of Prime type was significant (*t* = 8.05, *p* < 0.001); targets were named 41 ms faster when they were primed by identity words than by control words. This confirmed that low proficient bilinguals are able to process masked English primes sufficiently. The model also revealed a significant effect of Log subtlex frequency (*t* = –2.92, *p* < 0.01), and Length (*t* = 3.26, *p* < 0.01); higher frequency and shorter targets were associated with faster naming latency. The effect of Ortho_N was not significant (*t* < 1).

### Discussion

The critical result of Experiment 1 was that low-proficient Japanese–English bilinguals did not show an onset priming effect for L2-English targets (e.g., bark-BENCH = dark-BENCH). This finding differs from the significant onset priming effects typically found in reading aloud with native speakers of European languages (e.g., Forster and Davis, 1991; Kinoshita, 2000; Schiller, 2004) or the result obtained in Verdonschot et al. (2013) with proficient Chinese–English bilinguals. The low-proficient bilinguals, nevertheless, showed significant CV (mora) priming (bell-BENCH < cell-BENCH). In fact, the absence of onset priming together with the presence of CV (mora) priming parallel those reported by Verdonschot et al. (2011) with Japanese native speakers reading aloud Japanese kana and romaji-transcribed words. These data taken together suggest that the low-proficient bilinguals carried over their L1 unit to L2 word production.

In Experiment 2, high-proficient Japanese–English bilinguals were tested. Based on the results of Verdonschot et al. (2013) who found significant onset priming with proficient Chinese–English bilinguals, we expect to replicate that finding.

## Experiment 2: High-Proficient Japanese–English Bilinguals

### Methods

#### Participants

Forty-four highly proficient Japanese–English bilingual students from Waseda University (Tokyo, Japan) participated in the experiment for 1000 Yen (US$8). Their mean TOEIC score was 876 (range = 800–990) and they started studying English on average at the age of 9.9.

#### Stimuli

The stimuli were same as Experiment 1.

#### Apparatus and Procedure

These were identical to Experiment 1.

### Results

The data were analyzed identically to Experiment 1. For response latency analyses, the same outlier removal resulted in the removal of 0.3% of the data in the C/CV conditions, and 0.4% of the data in the Identity condition. In the identity priming condition, one item (DENY) was removed due to high error rates (>55%). Errors were analyzed identically to Experiment 1. However, again, error rates were generally very small, and there was no significant priming effect in any conditions, therefore, we only report the results of the response latency analyses. **Table 2** shows the mean RT and error rates for the three conditions.

**Table 2:** Mean naming latencies (ms) and percentage errors (%) for English targets primed by C primes, C control primes, CV primes, CV control primes, identity primes and identity control primes in Experiment 2, for high-proficient bilinguals.

	C condition	CV condition	Identity condition
Related prime	586 (3.9%)	563 (3.6%)	589 (6.1%)
Control prime	603 (2.8%)	584 (4.2%)	630 (7.3%)
Priming effect	17 (–1.1%)	21 (0.6%)	41 (1.2%)

#### Onset (C) and CV Priming Effects

As was the case in Experiment 1, Order did not significantly modulate the patterns of priming effects (*t*s < 1). As expected, the main effect of Order was significant (*t* = 7.95, *p* < 0.001), with targets being named significantly faster in the third than in the first block (note: Block 2 always contained identity primes, and therefore was not analyzed). The main effect of Prime Type was significant (*t* = 7.70, *p* < 0.001). There also was a significant effect of Overlap (*t* = –3.56, *p* < 0.001); across Prime Type, targets in the CV condition were named significantly faster than targets in the C condition. The two-way interaction between Overlap and Prime Type was marginally significant (*t* = 1.91, *p* = 0.064). Follow-up analyses of this marginal interaction revealed that high-proficient Japanese–English bilinguals showed a significant C (onset priming) effect (*t* = 3.71, *p* < 0.001, a 17 ms effect) as well as a significant CV priming effect (*t* = 6.63, *p* < 0.001, a 21 ms effect). The significant onset priming effect was consistent with the result of Verdonschot et al. (2013) with high-proficient Chinese–English bilinguals. As for the lexical characteristics of the targets, shorter naming latencies were associated with higher target frequency, (*t* = –7.73, *p* < 0.001), more orthographic neighbors (*t* = –2.60, *p* < 0.05) and shorter target length (*t* = 3.53, *p* < 0.001).

#### Identity Priming Effects

For response latency, as expected, the effect of Prime type was highly significant (*t* = 9.58, *p* < 0.001). Targets were named 41 ms faster when they were primed by identity words than by control words, again displaying the ability of bilinguals to efficiently process masked English primes. Among the target lexical characteristics, there was a significant effect of length (*t* = 3.08, *p* < 0.01) and a marginally significant effect of frequency (*t* = –1.94, *p* = 0.061). Shorter response latency was associated with higher target frequency and shorter target length. The effect of orthographic neighborhood size was not significant (*t* < 1).

### Discussion

Consistent with our prediction, highly proficient Japanese–English bilinguals showed a significant onset priming effect (17 ms) when reading aloud English words. This result suggested that high-proficient Japanese–English bilinguals employed a phoneme-sized proximate unit when producing L2 English words, although their L1 proximate unit is the mora (CV). The fact that the present results mirror those reported earlier with Chinese–English bilinguals (Verdonschot et al., 2013) strengthens the view that high-proficient bilinguals are able to use the proximate unit of the L2 language being spoken.

Both the low-proficient bilinguals (Experiment 1) and the high-proficient bilinguals (Experiment 2) showed a significant CV priming effect (bell-BENCH < cell-BENCH). This effect is not critical to our hypothesis (which concerns primarily the onset priming effect) and it could reflect mora priming (the basis on which we have expected low-proficiency bilinguals to show priming), or alternatively, it may reflect priming due to an overlap of two phonemic segments (initial C and V). It is not possible to determine *a priori* whether the CV priming effect observed with the high-proficient bilinguals reflects the usage of mora or phonemes. Nevertheless, there is one particular clue pointing toward the latter possibility, which is the fact that unlike the low-proficient bilinguals, the high-proficient bilinguals did not show statistically significantly greater priming due to an overlap in CV than C (onset) alone. This is consistent with the pattern that has been observed with monolingual speakers of English: an additional overlap in the vowel segment beyond the consonantal onset overlap leads to only a small increment in priming. For example, Kinoshita (2000) used 3-letter CVC non-word targets and reported that the onset priming effect (e.g., suf-SIB vs. muf-SIB) was substantial but an extra vowel overlap (sif-SIB) added only a statistically non-significant 3 ms increment; similarly, Mousikou et al. (2010) reported a small 4 ms (though statistically significant) increment.

In Experiment 3, we tested monolingual native speakers of English (i.e., a non-moraic language) using the same set of stimuli used in the preceding experiments. A successful demonstration of significant onset priming with native English speakers will further support the interpretation that high-proficient bilinguals (Experiment 2) used a phoneme-sized unit in producing the English words. Further, if the native English speakers show similar C and CV priming patterns as the high-proficient bilinguals, then such results will suggest that the high-proficient bilinguals’ CV priming effect was likely due to phonemic segmental overlap rather than mora-level overlap.

## Experiment 3: Monolingual Native English Speakers

### Methods

#### Participants

Forty-four monolingual native English speakers from Macquarie University (Sydney, Australia) participated in the experiment in return for course credit.

#### Stimuli

The same stimuli used in previous experiments were used.

#### Apparatus and Procedure

These were identical to the previous experiments.

### Results

The data were analyzed identically to Experiments 1 and 2. For response latency analyses, 0.1% of the data was removed as outliers in the C/CV conditions and also in the Identity condition. Errors were also analyzed identically to Experiment 1 and 2. However, again, error rates were generally very small, therefore, in what follows, we only report the results of response latencies. **Table 3** shows the mean RT and error rates for the three conditions.

**Table 3:** Mean naming latencies (ms) and percentage errors (%) for English targets primed by C primes, C control primes, CV primes, CV control primes, identity primes and identity control primes in Experiment 3, for monolingual English speakers.

	C condition	CV condition	Identity condition
Related prime	439 (2.0%)	432 (1.7%)	419 (3.0%)
Control prime	459 (3.7%)	459 (4.8%)	468 (5.4%)
Priming effect	20 (1.7%)	27 (3.1%)	49 (2.4%)

#### Onset (C) and CV Priming Effects

Again, the Order did not significantly affect the patterns of priming effects (*t*s < 1.68, *p*s > 0.10). The main effect of Order was significant (*t* = 2.05, *p* < 0.05) with faster naming latency in the third than in the first block. The main effect of Prime Type was significant (*t* = 11.19, *p* < 0.001). The effect of Overlap was not significant (*t* < 1). Similar to Experiment 2, there was a marginally significant interaction between Overlap and Prime Type (*t* = 1.94, *p* = 0.061). As expected, the follow-up interaction of the marginal interaction confirmed that there was a significant C priming effect (*t* = 8.67, *p* < 0.001, a 20 ms effect) as well as a significant CV priming effect (*t* = 8.52, *p* < 0.001 a 27 ms effect). Higher frequency targets were significantly associated with faster responding (*t* = –3.91, *p* < 0.001). Effects of target length or orthographic neighborhood size were not significant, both *t*s < 1.

#### Identity Priming Effects

There was a significant identity priming effect (*t* = 12.89, *p* < 0.001); targets primed by identity primes were named 49 ms faster than the same targets primed by unrelated primes. There was a significant effect of target frequency (*t* = –3.58, *p* < 0.001). There were no effects of ortho_N or Length (both *t*s < 1.1).

### Discussion

Monolingual native English speakers showed a significant onset priming effect, and a CV priming effect that did not differ in size (statistically) from the onset priming effect. This pattern mirrors that observed with the high-proficient bilinguals and contrasts with the low-proficient bilinguals (who showed a significant CV priming effect but not an onset priming effect). We take the results of Experiment 3 to suggest that the CV priming effect observed with the high-proficient bilinguals likely reflected an effect of phonemic segmental overlap.

## General Discussion

Previous studies have shown that the phonological unit used in word production differs across languages: for L1 English and Dutch speakers, the unit is suggested to be the phoneme (Levelt et al., 1999; Roelofs, 2015), for Chinese, the syllable (Chen et al., 2002; O’Seaghdha et al., 2010), and for Japanese, the mora (e.g., Kureta et al., 2006; Verdonschot et al., 2011; Verdonschot and Tamaoka, 2015). The current paper examined the phonological unit size used in L2 word production when bilinguals’ L1 and L2 languages employ different phonological unit sizes. The second, most essential, goal of this study was to investigate whether L2 English proficiency plays a role in the emergence of a phoneme-sized unit in English word production. To answer these two questions, we tested high- and low- proficient Japanese–English bilinguals in a masked priming read-aloud task. The results were clear: high-proficient bilinguals showed significant onset priming, but low-proficient bilinguals did not. The two groups of bilinguals, nevertheless, produced virtually identical identity and CV priming.

The results obtained with the low-proficient bilinguals – the absence of onset priming in the bilinguals whose first language is Japanese reading aloud English words - is important in establishing that the onset priming effect is not driven solely by the type of script (alphabetic letters). As noted, the original interpretation of masked onset priming effect was in terms of a serial letter-to-phoneme mapping process (Forster and Davis, 1991). The fact that low-proficient Japanese–English bilinguals do not show the onset priming effect indicates that reading aloud involves more than the mapping of letters to phonemes, and a full explanation of priming effects in reading aloud needs to take into account the processes involved in speech production.

Consistent with the assumption that the low-proficient bilinguals would use the phonological unit of their first language, the mora (CV), they showed no onset priming effect. In contrast, the high-proficient bilinguals showed an onset priming effect. These results suggest that highly proficient bilinguals seem to construct L2 English phonology similarly to native English speakers by incrementally inserting phonemes into the metrical frame.

**FIGURE 1:**
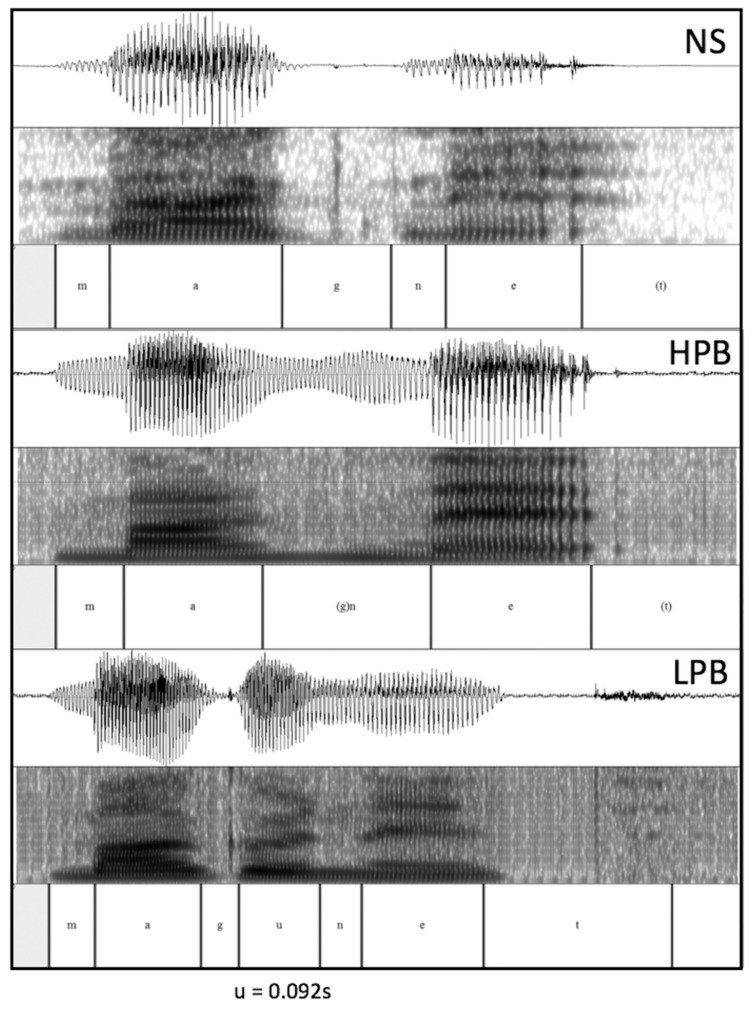
**Acoustic Waveforms for the word “magnet” (/’mæg nIt/) between a NS (Native Speaker), HPB (high-proficient bilingual) and LPB (low-proficient bilingual)**.

Additional support for the claim that low-proficient bilinguals used their L1 proximate unit (mora) to read aloud L2 words can be seen in the evidence of vowel insertions into a consonant cluster. **Figure 1** shows the acoustic waveforms for the word “magnet” produced by a native English speaker, a high-proficiency bilingual, and a low-proficiency bilingual speaker (all female). It can be seen that compared to native-speakers and high-proficient bilinguals (who do not insert vowels at g) this particular low-proficient bilingual is inserting an extra vowel in the word-medial consonant cluster, thereby changing the word structure from a disyllable to three (or possibly four) morae. Considering that the duration of a “real” vowel of this particular participant (“a” in “mAgnet”) is about 0.12 s, it seems reasonable to suggest that the “u” (∼0.092 s) is an epenthetic vowel with full insertion. We should point out that not all of our stimuli contained a consonant cluster, and also the likelihood of vowel insertion varies between consonant clusters (it is most evident for consonant clusters containing voiced stops). A more formal analysis of this phenomenon will therefore remain a topic for the future.

### What Aspects of L2 Proficiency are Responsible for the Use of an L2 Phonological Unit?

An obvious question that arises from the present study is *what aspect of proficiency* in L2 (English) is responsible for the shift in the proximate unit size used in L2 production. In the present experiments, the mean TOEIC score for highly proficient bilinguals was significantly higher than for low proficient bilinguals [mean for the highly proficient group = 876, low = 715, *t*(87) = 15.33, *p* < 0.001]. Our language history questionnaire, however, indicated that the two groups of bilinguals also differed in two other potentially important variables: (1) L2 AoA (the age at which the participant started learning English) [mean for the highly proficient group = 9.88, low = 11.53, *t*(87) = –2.94, *p* = 0.004); and (2) the number of months spent in an English-speaking country [mean for the highly proficient group = 21.20, low = 1.74, *t*(86) = 4.00, *p* < 0.001]. That is, our high proficient bilinguals started learning English significantly earlier and spent much longer in English speaking countries than low-proficient bilinguals did.

In order to find out which of the three factors: TOEIC (range 615–990), L2 AoA (range: 2–13), and Time spent in an English speaking country (range: 0–120 months) mostly contributed to the use of phoneme-size unit in speaking English words we analyzed the data from all bilinguals with a LME model, using the factors as continuous variables. Our initial analyses indicated that across all bilinguals, the three variables were correlated with each other: (1) TOEIC and the L2 AoA (*r* = –0.399, *p* < 0.001); (2) TOEIC and the Time spent (*r* = 0.511, *p* < 0.001); and (3) L2 AoA and Time spent, (*r* = –0.493, *p* < 0.001). In order to assess the unique predictive ability of each variable, the three factors were entered simultaneously in the model along with their respective interaction term with Prime Type, with the inverse RT as a dependent variable. All of the continuous variables were centered around their respective means.

The analysis revealed that the time spent in an English-speaking country significantly modulated onset priming (*t* = 2.23, *p* = 0.026), suggesting that the more time the participant spent in an English-speaking country, the greater the onset priming effect. Somewhat surprisingly, neither the TOEIC score nor the L2 AoA themselves uniquely explained the size of onset priming (both *t* < 1). This was also the case even when the effect of each variable was assessed individually (*t* = 2.30, *p* = 0.021 for the time spent in an English-speaking country, both *t*s < 1 for the TOEIC and L2 AoA).

Our analyses, therefore, showed that it was not the TOEIC score or L2 AoA, but it was the time spent in English speaking countries that contributed to the development of the phoneme-sized unit in L2 English production, Naturally, immersion in the L2 environment also leads to higher English proficiency as indicated by the significant relationship between TOEIC scores and the time spent in an English speaking country. The fact that the TOEIC score did not predict the onset priming effect is perhaps not too unexpected, given the test places greater emphasis on reading and listening comprehension rather than speech production. Thus to our question “what aspects of L2 proficiency are responsible for adopting an L2 proximate unit?”, a viable answer would be the extensive exposure to the L2 language environment (which is also associated with higher proficiency in L2). As this conclusion is based on a *post hoc* analysis, it needs to be corroborated in future studies using other indices that assess speech production ability more directly. However, from a practical point of view, the finding that the acquisition of the phoneme-sized phonological unit did not depend on L2 AoA is rather encouraging, as it suggests that the L2-specific proximate unit can be adopted by typical L2 learners of English residing in Japan who usually start learning English around the age of 10–13. Although acquisition of many aspects of phonology in a non-native language (e.g., accents) are suggested to be restricted by L2 AoA (e.g., Flege, 1988; Alario et al., 2010), the phonological encoding processes seem to be able to adapt their internal workings well after the L1 phonological unit size has been fully developed.

## Conclusion

O’Seaghdha et al. (2010) recently put forward the “proximate unit” principle, which suggests that the *initial phonological unit* used in the word-form encoding process differs across languages. Here we showed that when phonologically encoding English words, while low-proficient Japanese–English bilinguals use the phonological unit of their first language, namely the mora (CV), high-proficient bilinguals are able to use the phonological unit of the target language, namely the phoneme. Our data further showed that neither the L2 AoA or proficiency measured by standard tests of proficiency in English as a second language, but extensive exposure to its phonology seems to play a key role in the emergence of a phonological unit used in the construction of speech sounds in the second language.

A term frequently found in the psycholinguistic literature is the “Masked Onset Priming Effect” or MOPE (e.g., Schiller, 2008; Mousikou et al., 2010) which refers to the finding in Indo-European languages (such as English, Dutch) that faster speech onset latencies occur when reading aloud target words that are preceded by a prime sharing its onset with the target. However, it might be more reasonable to use the term “Masked Initial Segment Priming Effect” or *MISPE* instead as it has been shown that the effect may depend on the language at hand (e.g., the *onset* in Dutch/English, the *mora* in Japanese and the *syllable* in Chinese) as well as an individual’s proficiency level.

An issue that should be investigated in future studies is whether the present findings will be generalized to other tasks that are known to tap similar underlying phonological encoding processes (such as the form preparation paradigm). It will be also important to systematically examine how the development of phoneme-size units will affect various aspects of word processing in the L2 language (e.g., the ability to articulate a cluster of consonants without the vowel insertion, ability to manipulate phonemes, and so on).

## Author Contributions

All authors listed, have made substantial, direct and intellectual contribution to the work, and approved it for publication.

## Conflict of Interest Statement

The authors declare that the research was conducted in the absence of any commercial or financial relationships that could be construed as a potential conflict of interest.

## Footnotes

1 Hiragana and katakana are allographs, somewhat akin to the uppercase/lowercase distinction in the Roman alphabet. Katakana are used for foreign loan words and hiragana is used for words of Japanese origin and grammatical morphemes.

2 The TOEIC test is a paper-and-pencil, multiple-choice assessment developed and administered by Educational Testing Service (ETS). There are two separately timed sections of 100 questions each. It assesses a broad range of English skills (particularly reading and listening), especially in business settings. The test scores range from 10 to 990, with higher values indicating greater English proficiency. Many university students in Japan voluntarily take the test to quantify their English ability because many Japanese companies request to report TOEIC scores on their job applications.

3 As most moras found in Japanese consist of an onset and a nucleus (i.e., CV), the CV primes used in our experiments are functionally equivalent to a mora (especially for low proficient bilinguals).

4 In the analyses of errors, the initial model often failed to converge because of the complex specifications of random factors. In that case, we successively removed a random factor term until a model successfully converged.
