# Supplementary material for: The Emergence of a Phoneme-Sized Unit in L2 Speech Production: Evidence from Japanese–English Bilinguals
Source: Front Psychol. 2016 Feb 23;7:175. doi: 10.3389/fpsyg.2016.00175 (PMC4763048; doi:10.3389/fpsyg.2016.00175)
Supplement: Supplementary file 1 [file Data_Sheet_1.DOCX]

*Appendix –Stimuli for Experiment 1 and 2 (C + CV conditions)*

| Target | C Overlap | C Control | CV Overlap | CV Control |
| --- | --- | --- | --- | --- |
| BENCH | bark | dark | bell | cell |
| BITTER | bank | tank | bill | hill |
| BOTTLE | bat | rat | box | fox |
| BUDGET | boom | zoom | bump | jump |
| BUDGE | boat | coat | bust | lust |
| DESK | dust | rust | dead | head |
| DEPTH | dock | rock | deck | peck |
| DANCE | deal | seal | dad | bad |
| FABRIC | feed | seed | fan | ban |
| FANCY | fold | bold | fat | mat |
| FERRY | fire | hire | fence | hence |
| FISH | fake | lake | fit | hit |
| FINGER | folk | yolk | fix | mix |
| GANG | gold | mold | gap | lap |
| HANDLE | heal | meal | hack | lack |
| HEAVY | hide | ride | hell | yell |
| JACKET | joy | boy | jam | ham |
| LEVER | law | saw | leg | beg |
| LETTER | lint | hint | lend | bend |
| LIQUID | loss | moss | lip | hip |
| LISTEN | lone | tone | link | wink |
| LOCK | lag | tag | lot | pot |
| LUNCH | list | mist | luck | duck |
| MATCH | mop | top | mad | pad |
| MAGNET | mean | bean | map | rap |
| MIDDLE | mark | park | mill | pill |
| MINT | mall | fall | miss | kiss |
| MONSTER | math | path | mob | rob |
| PANTS | pea | tea | pack | tack |
| PITCH | pool | cool | pink | sink |
| PILLOW | poke | joke | pick | kick |
| RAPID | roll | poll | rack | sack |
| RISK | rain | pain | rip | dip |
| RICH | rub | pub | ring | sing |
| SEVEN | sit | kit | set | let |
| SINGLE | save | cave | silk | milk |
| SICK | sand | band | sip | tip |
| TEST | tall | wall | tell | fell |
| TEXT | toy | soy | tend | send |
| TAXI | tool | fool | task | mask |
| TICK | toss | boss | till | fill |
| WISH | wax | tax | wit | bit |

*Appendix (Continued) –Stimuli for Experiment 1 and 2 (Identity condition)*

| Target | Identity Overlap | Identity Control |
| --- | --- | --- |
| HATE | hate | dirt |
| WILD | wild | nose |
| CAMP | camp | wine |
| COLD | cold | live |
| COUNT | count | teach |
| GIFT | gift | male |
| GOAL | goal | pure |
| HURT | hurt | lane |
| WOLF | wolf | lent |
| KING | king | base |
| BULL | bull | lash |
| LOST | lost | hear |
| PAINT | paint | route |
| NAIL | nail | rude |
| NECK | neck | fort |
| BIRD | bird | sale |
| TEASE | tease | vowel |
| PUSH | push | code |
| ROSE | rose | fund |
| YARD | yard | bent |
| SOFT | soft | page |
| WOOD | wood | mine |
| PITY | pity | neon |
| BUSY | busy | hero |
| ZERO | zero | copy |
| DENY | deny | mama |
| TINY | tiny | papa |
| WAVE | wave | meat |
| NOISY | noisy | cable |
| REACT | react | solar |
| SORRY | sorry | limit |
| RIFLE | rifle | honor |
| MODEL | model | humor |
| PILOT | pilot | reply |
| CIVIL | civil | happy |
| FINAL | final | table |
| LABOR | labor | hotel |
| DOZEN | dozen | minor |
| MAGIC | magic | funny |
| MOVIE | movie | hurry |
| PENNY | penny | rigid |
| HABIT | habit | motor |
